# Retrospective evaluation of the effect of carotid artery stenosis on cerebral oxygen saturation during off-pump coronary artery bypasses grafting in adult patients

**DOI:** 10.1186/s12871-015-0164-z

**Published:** 2015-12-15

**Authors:** S. Toyama, K. Matsuoka, Y. Tagaito, M. Shimoyama

**Affiliations:** 1 Department of Anesthesiology, Medical Hospital of Tokyo Medical and Dental University, 1-5-45 Yushima, Bunkyo-Ku, Tokyo 113-8519 Japan; 2 Department of Anesthesiology, Teikyo University Chiba Medical Center, 3426-3 Anesaki, Ichihara-City, Chiba 299-0111 Japan; 3 Department of Palliative Medicine, Jikei University Graduate School of Medicine, 3-19-18 Nishi-shimbashi, Minato-Ku, Tokyo 105-8471 Japan

**Keywords:** Carotid artery stenosis, Cerebral tissue oxygen saturation, Coronary artery bypass grafting, Near-infrared spectroscopy

## Abstract

**Background:**

It is unknown whether cerebral oxygenation in patients with carotid artery stenosis (CAS) undergoing off-pump coronary artery bypass grafting (CABG) differs from that in patients without CAS. Thus, the effect of the presence of CAS ≥ 50 % on cerebral oxygenation during off-pump CABG in adult patients was evaluated retrospectively.

**Methods:**

Eleven patients with CAS ≥ 50 % and 14 patients without CAS ≥ 50 % were enrolled. Regional cerebral tissue oxygen saturation (rSO_2_) was quantified using near-infrared spectroscopy. Mean arterial pressure, cardiac index, central venous pressure (CVP), and rSO_2_ at specific points were collected, and significant changes in each parameter were detected using repeated analysis of variance. Mean rSO_2_ and minimum rSO_2_ during anastomosis were analyzed by one-way analysis of variance. Multiple logistic regression analysis was used to estimate the odds ratio (OR) with 95 % confidence interval (CI) for cerebral desaturation (a decrease in rSO_2_ ≥ 10 % from preoperative value).

**Results:**

Two patients with CAS ≥ 50 % who received complete carotid artery stenting preoperatively were excluded from the analyses. In both patients with and without CAS, a decrease in rSO_2_ and cardiac index and an increase in CVP were observed during anastomosis. Mean (SD) maximum decrease in rSO_2_ from preoperative value was 9.2 (12.7) % on the left side and 8.1 (11.7) % on the right side in patients with CAS ≥ 50 %, and 13.5 (11.3) % on the left side and 16.1 (9.8) % on the right side in patients without CAS ≥ 50 % (*p* = 0.316). Neurological complications were not identified in both patients with and without CAS ≥ 50 %. In multiple logistic regression analysis, CAS ≥ 50 % was not associated with an increased risk of cerebral desaturation (OR 0.160, 95 % CI 0.036–0.707, *p* = 0.016), and rSO_2_ decreased with decreasing cardiac index < 2.0 l/min/m^2^ (OR 3.287, 95 % CI 2.218–5.076, *p* < 0.001).

**Conclusions:**

CAS ≥ 50 % was not an independent risk factor of cerebral desaturation during off-pump CABG. Our results suggest that maintaining cardiac output can prevent a decrease in cerebral oxygenation in both patients with and without CAS ≥ 50 %.

## Background

Stroke following on-pump coronary artery bypass grafting (CABG) is a major source of morbidity and mortality [1]. Although the etiology of such stroke is multifactorial, the use of cardiopulmonary bypass (CPB) is a major contributor for brain injuries [2–4]. Hence, off-pump CABG is expected to reduce perioperative strokes compared with on-pump CABG [5, 6]. However, perioperative factors unrelated to CPB, such as advanced age, female sex, low left ventricular ejection fraction (LVEF), diabetes mellitus, chronic kidney disease, vascular disease and nonelective surgery, are demonstrated to be associated with stroke following CABG [7]. Thus, the superiority of off-pump CABG to on-pump CABG for prevention of perioperative stroke is controversial [8–13].

Four to 17 % of patients undergoing CABG have carotid artery stenosis (CAS) ≥ 50 % [14, 15], and concomitant CAS is associated with an increased risk of stroke following on-pump CABG [16–18]. Since cerebral hemodynamics is impaired depending on the degree of the stenosis [19], CAS can provoke cerebral oxygen supply–demand mismatch when hemodynamic impairment occurs during surgery. However, the pathophysiological mechanism of most postoperative ischemic strokes in patients with CAS undergoing on-pump CABG is thought to be microemboli from ascending aorta and/or carotid artery, or cardioembolism rather than hypoperfusion to cause cerebral oxygen supply–demand mismatch [17, 20]. Meanwhile, in general, hemodynamic depression due to moving the heart from its natural position during anastomosis can lead to a decrease in cerebral oxygenation more frequently during off-pump CABG than on-pump CABG [21]. Thus, CAS is likely to be associated with an increased risk of cerebral oxygen supply–demand mismatch to cause cerebral ischemia during off-pump CABG. However, little information is available for the effect of the presence of CAS on cerebral oxygenation in patients undergoing off-pump CABG.

Near-infrared spectroscopy (NIRS) is a non-invasive measure that provides continuous monitoring of regional cerebral tissue oxygen saturation (rSO_2_), and rSO_2_ values reflect hemoglobin saturation in a mixture of arteries, capillaries, and veins [22]. Although most NIRS devices assume that the hemoglobin content of the cerebral cortex is distributed 75 % in the venous and 25 % in the arterial district, a previously reported study shows that there are considerable biological variations in individual cerebral arterial-venous ratios between patients, suggesting that absolute rSO_2_ values present inter-individual variability [23]. However, using NIRS as a trend monitor can minimize the inter-individual variables, and NIRS has been demonstrated to be a clinically useful monitor of cerebral oxygenation in various settings [24–30]. Thus, we retrospectively examined the effect of CAS ≥ 50 % on cerebral oxygenation during off-pump CABG in adult patients by assessing rSO_2_ values derived from NIRS.

## Methods

### Patients

Following the Institutional Ethics Committee approval of Teikyo University, Tokyo, Japan (reference number 13–252), we retrospectively analyzed data from patients who underwent off-pump CABG at Teikyo University Chiba Medical Center (Chiba, Japan) from April 2009 to March 2014. All patients received carotid duplex sonography and brain computed tomography (CT) preoperatively, and the severity of CAS was assessed using area stenosis as stenosis (stenosis ≥ 50 %) or none (stenosis < 50 %). Patients who received mechanical ventilation under intubation preoperatively and did not receive pulmonary artery catheterization and rSO_2_ monitoring during surgery were excluded from the present study. All operations were performed by the same cardiovascular surgical team. When preoperative thoracic enhanced CT and intraoperative transaortic echocardiography revealed calcifications in the ascending aorta, an aortic no-touch technique, in which only arterial grafts without surgical manipulation of the ascending aorta were used, or great saphenous vein graft anastomosis to the ascending aorta using the aortic proximal anastomotic device was performed.

### Anesthetic technique

On the morning of surgery, patients were allowed to take their routine medication, except for angiotensin-converting enzyme inhibitors and angiotensin-II receptor blockers. Standard monitoring with electrocardiography, pulse oximetry, end-tidal carbon dioxide (ETCO_2_), bispectral index (BIS), invasive arterial pressure measurement and rectal temperature measurement was performed. Arterial pressure was recorded continuously via the right radial artery catheter which was inserted before induction of anesthesia. Cerebral oxygenation was monitored by measuring rSO_2_ values derived from an INVOS 5100C cerebral oximeter (INVOS, Covidien, Mansfield, MA). Two disposable NIRS sensors were applied on each side of the forehead for continuous monitoring of rSO_2_ of the corresponding brain hemisphere. Anesthesia was induced with a continuous infusion of remifentanil (0.3–0.5 μg/kg/min) and target controlled infusion of propofol (2 μg/ml), and a bolus of rocuronium (1 mg/kg). The lungs were ventilated mechanically with oxygen enriched air (fractional inspired oxygen of 0.6) adjusted to keep ETCO_2_ between 35 and 40 mmHg. The remifentanil infusion was titrated in a range of 0.2–0.5 μg/kg/min to the patients’ clinical requirement as judged by the anesthesiologist present. The propofol infusion was titrated to keep BIS level between 40 and 50. After tracheal intubation, a transesophageal echocardiography probe was placed, and pulmonary artery catheter was inserted via the right internal jugular vein. Central venous pressure (CVP), pulmonary artery pressure, cardiac index, and mixed venous blood oxygen saturation (SvO_2_) were recorded continuously. Cardiac output was measured by thermodilution technique with a pulmonary artery catheter, and the cardiac index was calculated.

Crystalloid was infused starting before the induction of anesthesia until the completion of surgery. An initial heparin dose of 300 IU/kg was administered after pericardiotomy. Protamine 1.5 mg/kg was administered after anastomosis. When hemodynamic status was unstable (cardiac index < 2.0 l/min/m^2^ and/or mean arterial pressure (MAP) < 60 mmHg) despite optimization of the circulating volume, dopamine was administered to increase CI above 2.0 l min/m^2^, and phenylephrine or norepinephrine was administered to increase MAP above 60 mmHg. All patients received a continuous infusion of nicorandil (2 mg/hr) throughout surgery. Blood transfusion was performed to maintain hemoglobin between 9 and 10 g/dl. Rectal temperature was kept between 36 and 37 °C.

After anastomosis, fentanyl was given, and remifentanil was discontinued prior to the completion of surgery. Propofol administration was continued until postoperative tracheal extubation in the intensive care unit.

### Perioperative data collection

Baseline rSO_2_ values and hemodynamic data (heart rate and MAP) were obtained before induction of anesthesia while patients were breathing under room air. Values of MAP, cardiac index, CVP, and rSO_2_ were continuously measured and simultaneously recorded at the same time points (every 5 min) until the completion of surgery. Since the duration of anesthesia varied with cases due to the differences of the number of anastomosis, the time course comparison of these data between the two groups was performed at specific points during surgery: at skin incision, at heparin administration, at the beginning of grafting the left anterior descending coronary artery (LAD), at the beginning of grafting the left circumflex coronary artery (LCX), at the beginning of grafting the right coronary artery (RCA), at protamine administration, and at chest closure. Although an absolute rSO_2_ value for cerebral ischemia prediction cannot be defined, a decrease in rSO_2_ ≥ 10 % from baseline value indicates cerebral desaturation associated with cerebral dysfunction [22], and a decrease in rSO_2_ ≥ 20 % from baseline value indicates a critical reduction in cerebral oxygenation and perfusion [25, 27, 28]. Thus, the rSO_2_ values were also normalized by expressing them as a percentage change from baseline value.

Postoperative complications including major adverse cardiovascular events [31], neurological complications, duration of mechanical ventilation, and early postoperative death (<30 days) were compared between patients with and without CAS. Neurological complication was defined as focal neurologic deficit persisting ≥ 24 h and confirmed by brain CT or magnetic resonance imaging (MRI).

### Statistical analysis

We did not have preliminary data and similar previously reported studies to perform a power analysis. The sample size of a paired *t*-test was indicated to be n = 10 (α = 0.05, β = 0.20) to reveal a significant decrease in rSO_2_ value from preoperative value during surgery in patients with CAS, assuming that mean (SD) relative change between minimum and preoperative rSO_2_ was 10 (10) %, which was based on a previously reported study showing that the decrease in rSO_2_ from preoperative value of 5–14 % was observed during carotid cross-clamping in patients undergoing carotid endarterectomy with general anesthesia and ventilation adjusted to fractional inspired oxygen of 0.3–1.0 [32]. Patient characteristics and perioperative data were analyzed by unpaired Student’s *t*-test, Mann–Whitney *U*-test or Fischer’s exact probability test, one-way analysis of variance (ANOVA) with the Dunn *post hoc* test, as appropriate. Spearman’s rank correlation coefficient (*r*) was calculated to examine the relationships among preoperative patient characteristics (the degree of CAS or hemodynamic data (preoperative LVEF and baseline MAP) and rSO_2_ value. Hemodynamic data (MAP, cardiac index, and CVP) and rSO_2_ at specific points during surgery in patients with and without CAS were analyzed by two-way repeated ANOVA with the Bonferroni *post hoc* test. In two-way repeated ANOVA, missing data were imputed using an expectation-maximization algorithm. In patients with CAS and without CAS, mean rSO_2_ value, minimum rSO_2_ value, and the duration of a decrease in rSO_2_ ≥ 10 % and ≥ 20 % from baseline value were analyzed by one-way ANOVA with the Dunn *post hoc* test. Multiple logistic regression analysis was used to estimate the odds ratio (OR) with 95 % confidence interval (CI) for cerebral desaturation (a decrease in rSO_2_ ≥ 10 % from baseline value) during anastomosis, with independent factors of MAP < 60 mmHg, cardiac index < 2.0 l/min/m^2^, CVP > 10 mmHg, CAS, CAS side of the forehead, and bilateral CAS. The ORs for cerebral desaturation were adjusted for every 1 μg/kg/min increase in dopamine and every 0.01 μg/kg/min increase in noradrenaline. Statistical significant was defined as *p* < 0.05. The SigmaPlot statistical software package for Windows (version 11.2, Systat, Sa Jose, CA, USA) was used for statistical analysis.

## Results

**Fig. 1:**
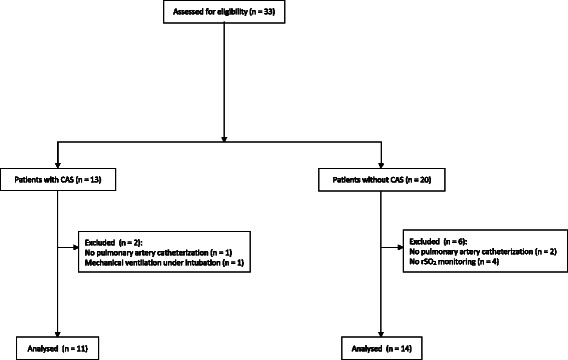
CONSORT flow diagram of the patients included in the study. rSO_2_ = regional cerebral tissue oxygen saturation

Figure 1 shows the flow diagram for the study. Thirty three patients were enrolled in the present study, and 8 subjects were excluded from final analysis because of the following factors: mechanical ventilation under midazolam administration (1 patient with CAS); no use of pulmonary artery catheterization (1 patient with CAS and 2 patients without CAS); no use of NIRS (4 patients without CAS). As a result, data analysis was performed on 25 patients (11 patients with CAS and 14 patients without CAS).

The demographic data in patients with CAS are presented in Table 1. Four patients had a unilateral high-grade (≥70 %) CAS, 1 patient had a unilateral moderate-grade (≥50 %) CAS with a contralateral occlusion, 2 patients had bilateral high-grade CAS, 2 patients had a unilateral high-grade CAS with a contralateral moderate-grade CAS, and 2 patients had bilateral moderate-grade CAS. In 3 of 9 patients with high-grade CAS, preoperative single photon emission computed tomography (SPECT) with acetazolamide stress was performed, but 6 other patients with high-grade CAS received CABG without preoperative SPECT with acetazolamide stress since the symptom of angina pectoris was uncontrollable by medical therapy. Three patients received carotid artery stenting prior to CABG. However, carotid artery stenting was unsuccessful in 1 patient. Thus, 2 patients with CAS who received complete carotid artery stenting preoperatively were excluded from the analyses.

**Table 1:** Demographic information and baseline regional cerebral tissue oxygen saturation (rSO_2_) in patients with carotid artery stenosis (CAS)

Case	Age (yr)	CAS (Left/Right) (%)	Preoperative SPECT	Preoperative stenting	Baseline rSO_2_ (Left/Right) (%)	Preoperative LVEF (%)	Baseline MAP (mmHg)
1	50	0/80	Yes	Yes	72/72	61	117
2	50	75/50	Yes	No	52/51	52	77
3	77	50/50	No	No	71/71	64	113
4	80	50/100	Yes	Incomplete	56/53	58	103
5	68	50/50	No	No	65/72	41	97
6	45	0/80	No	Yes	59/54	20	100
7	52	75/70	No	No	48/50	22	67
8	68	88/50	No	No	70/66	58	95
9	78	70/70	No	No	61/51	42	82
10	65	90/0	No	No	52/57	60	93
11	71	80/0	No	No	66/70	67	75

*LVEF* left ventricular ejection fraction, *SPECT* single photon emission computed tomography with acetazolamide stress, *MAP* mean arterial pressure

The demographic and preoperative clinical findings of the study patients are shown in Table 2. There were no significant differences between patients with and without CAS except for a history of cerebral infarction and/or transient ischemic attack (TIA) (9/9 [100.0 %] and 2/14 [14.3 %], respectively, *p* < 0.001). In patients with CAS, baseline rSO_2_ did not correlate with the degree of CAS (*r* = −0.376, *p* = 0.120) but correlated with both preoperative LVEF (*r* = 0.509, *p* = 0.0306) and baseline MAP (*r* = 0.572, *p* = 0.0129). Meanwhile, in patients without CAS, baseline rSO_2_ correlated with neither preoperative LVEF (*r* = 0.153, *p* = 0.433) nor baseline MAP (*r* = 0.275, *p* = 0.154).

**Table 2:** Preoperative demographic data in patients with and without carotid artery stenosis (CAS)

	Patients with CAS (n = 9)	Patients without CAS (n = 14)	*P*-value
Age (yr)	67 [11]	69 [7]	0.797
Gender (Male/Female)	8/1	9/5	0.340
Body mass index (kg/m^2^)	22 [2]	23 [4]	0.092
Preoperative complications (n)			
Cerebral infarction and/or TIA	9 [100.0%]	2 [14.3%]	<0.001
Hypertension	7 [77.8%]	12 [85.7%]	1.000
Diabetes mellitus	7 [77.8%]	8 [57.1%]	0.659
Hyperlipidemia	2 [22.2%]	3 [21.4%]	1.000
Chronic kidney disease on hemodialysis	2 [22.2%]	3 [21.4%]	1.000
Congestive heart failure	2 [22.2%]	0 [0.0%]	0.142
Atrial fibrillation	0 [0.0%]	0 [0.0%]	–
Preoperative cardiac evaluation			
Left ventricular ejection fraction (%)	58 [22 – 67]	57 [31 – 74]	0.387
Mitral regurgitation (grade*)	0 [0 – 3]	0 [0 – 3]	0.607
Aortic regurgitation (grade*)	0 [0 – 2]	0 [0 – 2]	0.823
Preoperative hemodynamic data			
HR (beats/min)	76 [9]	70 [11]	0.181
MAP (mmHg)	89 [15]	96 [15]	0.293
Preoperative rSO_2_ (Left/Right) (%)	60 [8]/60 [9]	63 [10]/65 [9]	0.422
Preoperative hemoglobin (g/dl)	11.0 [0.8]	10.6 [1.4]	0.488

Data are mean [SD], number, number [proportion] or median [range]

*TIA* transient ischemic attack, *HR* heart rate, *MAP* mean arterial pressure, *rSO*_2_ regional cerebral tissue oxygen saturation *Grade of regurgitation: 0 = none, 1 = trivial, 2 = mild, 3 = moderate, 4 = severe

Surgical-related data and postoperative outcomes are presented in Table 3. Aortic not-touch technique was used more frequently in patients with CAS than those without CAS (4/9 [44.4 %] and 0/14 [0.0 %], respectively, *p* = 0.014), but the frequency of the use of aortic no-touch technique or aortic proximal anastomotic device was not different between patients with CAS and those without CAS (5/9 [55.6 %] and 2/14 [14.3 %], respectively, *p* = 0.066). Postoperative morbidity and mortality did not differ between patients with and without CAS, and neurological complications were not identified in both patients with and without CAS.

**Table 3:** Intraopeative and postoperative clinical data in patients with and without carotid artery stenosis (CAS)

	Patients with CAS (n = 9)	Patients without CAS (n = 14)	*P*-value
Surgical-related data			
Anesthesia time (min)	466 [119]	425 [67]	0.301
Operation time (min)	347 [89]	335 [64]	0.707
Duration of anastomosis (min)	170 [45]	186 [52]	0.455
Number of anastomosis (n)	3 [1 – 5]	4 [2 – 4]	0.341
Aortic no-touch technique (n)	4 [44.4%]	0 [0.0%]	0.019
Aortic proximal anastomosis device (n)	1 [11.1%]	2 [14.3%]	1.000
Packed red blood cell transfusion (U)	0 [0 – 6]	2 [0 – 6]	0.224
Cumulative dose of phenylephrine (μg)	400 [0 – 650]	150 [0 – 800]	0.948
Maximum dose of dopamine (μg/kg/min)	5 [0 – 5]	3 [0 – 5]	0.316
Maximum dose of norepinephrine (μg/kg/min)	0.15 [0.03 – 0.5]	0.1 [0.0 – 0.25]	0.159
Postoperative outcomes			
Mechanical ventilation (day)	1 [1 – 1]	1 [0 – 4]	0.667
Major adverse cardiovascular events (n)			
Acute coronary event	0 [0.0%]	1 [7.1%]	1.000
Congestive heart failure	2 [22.2%]	0 [0.0%]	0.142
Arrhythmia	1 [11.1%]	0 [0.0%]	0.391
Neurological complications	0 [0.0%]	0 [0.0%]	-
Early postoperative death (< 30 days)	0 [0.0%]	0 [0.0%]	-

Data are mean [SD] or median [range]

**Fig. 2:**
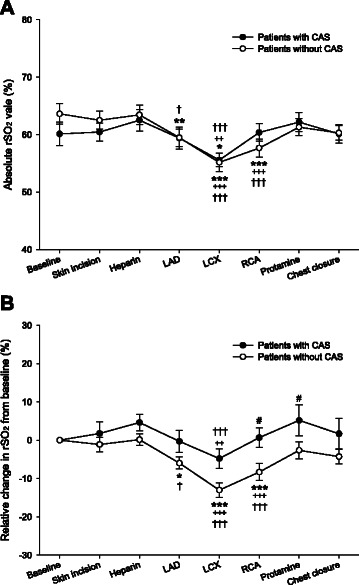
Regional cerebral tissue oxygen saturation (rSO_2_) in patients with and without carotid artery stenosis (CAS) (A: Absolute rSO_2_ value, B: Relative change from baseline value). The data are presented as mean (SE) and at specific points during anesthesia: before anesthesia induction (Baseline), at skin incision (Skin incision), at heparin administration (Heparin), at the beginning of grafting the left anterior descending coronary artery (LAD), at the beginning of grafting the left circumflex coronary artery (LCX), at the beginning of grafting the right coronary artery (RCA), at protamine administration (Protamine), and at chest closure (Chest closure). *p < 0.05, **p < 0.01, ***p < 0.001 compared with the group’s Baseline. ++*p* < 0.01, +++*p* < 0.001 compared with the group’s Skin incision. †*p* < 0.05, ††*p* < 0.01, †††*p* < 0.001 compared with the group’s Heparin administration. #*p* < 0.05 compared with patient without CAS

Figure 2 shows absolute rSO_2_ values or relative changes in rSO_2_ from baseline value at specific points during surgery in the study patients. Two-way repeated ANOVA revealed a significant within-subjects effect in both an absolute rSO_2_ value and a relative change in rSO_2_ from baseline value (*p* < 0.001 and *p* < 0.001, respectively). An absolute rSO_2_ value did not differ between patients with and without CAS (*p* = 0.805), but a relative changes in rSO_2_ from baseline value was smaller in patients with CAS than patients without CAS (*p* = 0.048).

Table 4 shows rSO_2_ values during anastomosis in patients with CAS. The degree of CAS correlated with neither mean relative change in rSO_2_ from baseline value (*r* = 0.327, *p* = 0.181) nor relative change in minimum rSO_2_ from baseline value (*r* = 0.424, *p* = 0.0774). Table 5 shows rSO_2_ values during anastomosis in the study patients. Mean (SD) maximum decrease in rSO_2_ from preoperative value was 9.2 (12.7) % on the left side and 8.1 (11.7) % on the right side in patients with CAS, and 13.5 (11.3) % on the left side and 16.1 (9.8) % on the right side in patients without CAS (*p* = 0.316). The duration of a decrease in rSO_2_ ≥ 10 % and ≥ 20 % from baseline value during anastomosis did not differ between patients with and without CAS (*p* = 0.479 and *p* = 0.394, respectively). The incidence of a decrease in rSO_2_ ≥ 20 % from baseline value during anastomosis was identified on both the sides of the forehead in one patient with CAS (11.1 %) and on the 5 left and 3 right sides of the forehead in 5 patients without CAS (35.7 %) (*p* = 0.340).

**Table 4:** Regional cerebral tissue oxygen saturation (rSO_2_) during anastomosis in patients with carotid artery stenosis (CAS)

	Left side				Right side			
Case	CAS (%)	Baseline rSO_2_ (%)	Mean rSO_2_ (%)	Minimum rSO_2_ (%)	CAS (%)	Baseline rSO_2_ (%)	Mean rSO_2_ (%)	Minimum rSO_2_ (%)
2	75	52	58	53	50	51	54	51
3	50	71	65	60	50	71	65	60
4	50	56	53	48	100	53	52	48
5	50	65	52	45	50	72	59	54
7	75	48	53	48	70	50	56	50
8	88	70	74	60	50	66	66	53
9	70	61	57	55	70	51	57	53
10	90	52	62	59	0	57	65	62
11	80	66	64	57	0	70	68	59

**Table 5:** Regional cerebral tissue oxygen saturation (rSO_2_) during anastomosis

	Patients with CAS (n = 9)		Patients without CAS (n = 14)		*P*- value
	Left side	Right side	Left side	Right side	
Baseline rSO_2_ (%)	60 [8]	60 [9]	63 [10]	65 [9]	0.422
During anastomosis					
Mean rSO_2_ (%)	60 [7]	60 [6]	59 [8]	60 [8]	0.981
Mean relative change in rSO_2_ from baseline (%)	0.6 [12.3]	1.6 [10.5]	-4.8 [9.2]	-7.3 [7.2]	0.100
Minimum rSO_2_ (%)	54 [6]	54 [5]	54 [8]	54 [8]	0.977
Relative change in minimum rSO2 from baseline (%)	-9.2 [12.7]	-8.1 [11.7]	-13.5 [11.3]	-16.1 [9.8]	0.316
A decrease in rSO_2_ ≥ 10% from baseline (min)	0 [0 – 75]	30 [0 – 75]	23 [0 – 195]	30 [0 – 165]	0.479
A decrease in rSO_2_ ≥ 20% from baseline (min)	0 [0 – 45]	0 [0 – 45]	0 [0 – 120]	0 [0 – 120]	0.394
Absolute rSO_2_ < 40 % (n)	0	0	0	0	–

Data are mean [SD] or median [range]

*CAS* carotid artery stenosis

**Fig. 3:**
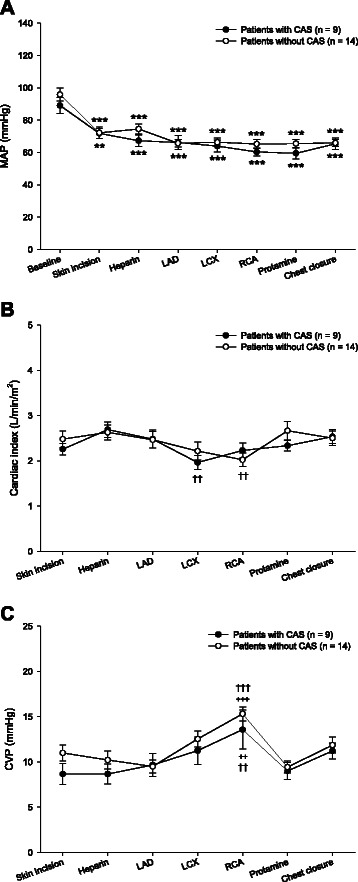
Hemodynamic data in patients with and without carotid artery stenosis (CAS) (A: Mean arterial pressure (MAP), B: Cardiac index, C: Central venous pressure (CVP)). The data are presented as mean (SE) and at specific points during anesthesia: before anesthesia induction (Baseline), at skin incision (Skin incision), at heparin administration (Heparin), at the beginning of grafting the left anterior descending coronary artery (LAD), at the beginning of grafting the left circumflex coronary artery (LCX), at the beginning of grafting the right coronary artery (RCA), at protamine administration (Protamine), and at chest closure (Chest closure). **p < 0.01, ***p < 0.001 compared with the group’s Baseline or Skin incision. ++*p* < 0.01, +++*p* < 0.001 compared with the group’s Skin incision. ††*p* < 0.01, †††*p* < 0.001 compared with the group’s Heparin administration.

Hemodynamic data at specific points during surgery are presented in Fig. 3. In both patients with and without CAS, significant within-subjects effects were detected for MAP (*p* < 0.001), cardiac index (*p* < 0.001) and CVP (*p* < 0.001). Decreases in cardiac index and increases in CVP were observed at the positioning of the heart for grafting LCX and/or RCA. However, there were no significant differences in MAP (*p* = 0.245), cardiac index (*p* = 0.620), and CVP (*p* = 0.330) between patients with and without CAS.

The results of multiple logistic regression analysis for cerebral desaturation during anastomosis are presented in Table 6. CAS was not associated with an increased risk of cerebral desaturation (OR 0.160, 95 % CI 0.036–0.707, *p* = 0.016). Cardiac index < 2.0 l/min/m^2^ was associated with an increased risk of cerebral desaturation (OR 3.287, 95 % CI 2.128–5.076, *p* < 0.001), while neither MAP < 60 mmHg nor CVP > 10 mmHg was associated with an independent increased risk of cerebral desaturation (OR 1.011, 95 % CI 0.623–1.640, *p* = 0.965 and OR 1.483, 95 % CI 0.965–2.279, *p* = 0.072, respectively).

**Table 6:** Multiple logistic regression analysis for a decrease in regional cerebral tissue oxygen saturation (rSO_2_) ≥ 10 % from baseline value (cerebral desaturation)

	OR	95 % CI	Coefficient	SE	*P*-value
MAP < 60 mmHg	1.011	0.623 – 1.640	0.011	0.247	0.965
Cardiac index < 2.0 l/min/m^2^	3.287	2.128 – 5.076	1.190	0.222	<0.001
CVP > 10 mmHg	1.483	0.965 – 2.279	0.394	0.219	0.072
CAS	0.160	0.036 – 0.707	−1.830	0.757	0.016
CAS side of the forehead	0.476	0.040 – 5.670	−0.741	1.264	0.557
Bilateral CAS	2.565	0.310 – 21.20	0.942	1.078	0.382
Dopamine	1.474	1.228 – 1.770	0.388	0.093	<0.001
Noradrenaline	1.020	0.981 – 1.060	0.020	0.020	0.315

Odds ratios (ORs) with 95 % confidence interval (CI) for cerebral desaturation during off-pump coronary artery bypass grafting for mean arterial pressure (MAP) < 60 mmHg, cardiac index < 2.0 l/min/min^2^, central venous pressure (CVP) > 10 mmHg, carotid artery stenosis (CAS), CAS side of the forehead, bilateral CAS, every 1 μg/kg/min increase in a dose of dopamine, and every 0.01 μg/kg/min increase in a dose of noradrenaline

## Discussion

In the present study, cerebral oxygenation significantly decreased during anastomosis in both patients with and without CAS. However, the mean rSO_2_ value and maximum decrease in rSO_2_ value during anastomosis were not different between patients with and without CAS, and CAS was not a significant independent risk factor of a decrease in rSO_2_ ≥ 10 % from preoperative value. Moreover, a decrease in rSO_2_ during anastomosis was more strongly associated with a decrease in cardiac index rather than a decrease in MAP and an increase in CVP in both patients with and without CAS.

Since hemodynamic depression during cardiac displacement can lead to a decrease in cerebral oxygenation more frequently during off-pump CABG than on-pump CABG [21], patients with CAS, whose cerebral hemodynamic is impaired depending on the degree of stenosis [19], is likely to be associated with an increased risk of cerebral oxygen supply–demand mismatch to cause cerebral ischemia during off-pump CABG. However, it is unknown whether the presence of CAS can affect cerebral oxygenation in patients undergoing off-pump CABG. Consistent with previously reported studies in patients without cerebrovascular disease undergoing off-pump CABG [27, 33], rSO_2_ value decreased during anastomosis in both patients with and without CAS. However, there were no significant differences in mean relative change in rSO_2_ from preoperative value and maximum decrease in rSO_2_ value from preoperative value during anastomosis between patients with and without CAS, and the incidence of a critical reduction in rSO_2_ was not different between patients with and without CAS. Moreover, multiple logistic regression analysis demonstrated that the presence of CAS did not increase a risk of a decrease in rSO_2_ ≥ 10 % from preoperative value, which indicates cerebral desaturation associated with cerebral dysfunction [22], and postoperative neurological complications were not identified in both patients with and without CAS. Perioperative stroke is reported to occur in 1.0–11.1 % of patients after off-pump CABG [12, 13, 34–37]. However, studies showing the incidence of strokes in patients with CAS after off-pump CABG are limited [35, 37]. Although a recently reported study demonstrated that CAS ≥ 50 % was an independent predictor of postoperative stroke or TIA in patients receiving off-pump CABG [35], most of postoperative stroke or TIA in the study occurred several days after the surgery. This suggested that the postoperative neurological complications were related to embolus associated with postoperative hypercoagulation and atrial fibrillation rather than to intraoperative cerebral ischemia. In addition, the degree of CAS has not been shown to be associated with a risk of perioperative stroke after noncardiac surgery [38]. In the present study, although the degree of CAS varied from 50 % to 100 %, there were no significant correlation between the degree of CAS and changes in rSO_2_ values during anastomosis. Considering these findings together with our results, CAS does not likely seem to be an independent risk factor of cerebral ischemia during off-pump CABG. Furthermore, in both patients with and without CAS, changes in rSO_2_ during anastomosis did not differ between the left and right side of the forehead, and multiple logistic regression analysis showed that bilateral CAS was not associated with an increased risk of a decrease in rSO_2_ ≥ 10 % from preoperative value. Although patients with bilateral CAS > 70 % has been shown to be an independent risk factor of early acute cerebrovascular complications following off-pump CABG [37], our results may suggest that cerebral bi-hemispheric perfusion by Willis circle was working properly in all of the study patients.

In the present study, preoperative rSO_2_ in patients with CAS correlated with both preoperative LVEF and MAP, while preoperative rSO_2_ in patients without CAS correlated with neither preoperative LVEF nor MAP. These results suggest that cerebral oxygenation in patients with CAS depended on hemodynamic status more strongly compared with patients without CAS. However, changes in rSO_2_ values after the induction of anesthesia did not differ between patients with and without CAS under the same anesthetic and hemodynamic management. Anesthesia-induced suppression of neural activity decreases cerebral metabolic rate of oxygen CMRO_2_ [39], and cerebral oxygenation during anesthetic-induced hypotension is likely to be maintained due to the neurovascular coupling between cerebral blood flow (CBF) and CMRO_2_ [40]. The neurovascular coupling is preserved even in patients with head injuries during propofol anesthesia [41]. Moreover, under general anesthesia, cerebral oxygenation has been shown to be maintained at preoperative level by increasing inspired oxygen fraction in both patients with and without CAS [32, 42, 43]. Thus, in both patients with and without CAS, unchanged rSO_2_ values after the induction of anesthesia may indicate that cerebral oxygen supply–demand mismatch was not caused by anesthetic-induced hypotension. On the contrary, in both patients with and without CAS, a decrease in cardiac index and an increase in CVP were observed during grafting the LCX and/or RCA. Multiple logistic regression analysis showed that a decrease in cardiac index, but not an increase in CVP, was associated with an increased risk of a decrease in rSO_2_ ≥ 10 % from preoperative value. Several studies have demonstrated that an increase in cardiac output can cause an increase in cerebral oxygenation and perfusion [27, 43–48], and cardiac output is likely to influence CBF independent of cerebral autoregulation [27, 49]. In addition, cerebral oxygenation is shown to be largely maintained by increased extracerebral perfusion due to increased cardiac output [50]. Since decreased CBF was compensated for increased cerebral blood volume due to recruitment of collateral pathway from extracerebral artery in patients with cerebrovascular disease [19], in patients with CAS as well as those without CAS, the influence of changes in cardiac output on cerebral oxygenation is likely to be large. Meanwhile, since NIRS measures arterial, venous and capillary oxygen saturation, an increase in CVP following cardiac displacement can cause cerebral venous congestion and lead to a decrease in rSO_2_ value. However, patients were usually positioned in a Trendelenburg position during grafting the LCX and/or RCA. Several studies have shown no relationship between the Trendelenburg position and a change in rSO_2_ value since an increase in CBF due to the Trendelenburg position increases oxygen delivery [51, 52]. Thus, in the present study, increased CVP might not be an independent risk factor of a decrease in rSO_2_ ≥ 10 % from baseline value. In addition, caution is needed in rSO_2_ monitoring during administration of potent vasoconstrictors. Noradrenaline and phenylephrine have been demonstrated to increase internal carotid artery flow, but decrease extracerebral blood flow, resulting in decreased rSO_2_, without affecting CBF [50, 53–55]. The lack of relationship between a decrease in rSO_2_ ≥ 10 % from preoperative value and a decrease in MAP in the present study patients may reflect such effect of vasoconstrictors on rSO_2_ values derived from NIRS.

Limitations of the present study include its retrospective nature, small sample size for determining the significant differences in rSO_2_ values between patients with and without CAS, and the lack of postoperative cognitive dysfunction assessment. Although our results suggested that CAS was not an independent risk factor of cerebral desaturation during off-pump CABG, the OR of CAS of 0.16 (95 % CI, 0.036–0.707) for cerebral desaturation also means that the risk of cerebral desaturation was lower in patients with CAS than those without CAS. All of the study patients were managed under the same anesthetic protocol, in which rSO_2_ target value for preventing cerebral ischemia was not determined, but surgeons and anesthesiologists might have paid more attention to hemodynamics and oxygen delivery in patients with CAS. Thus, the OR might be affected by a bias related to the retrospective nature of the study. Surgical and anesthetic management in the present study were performed by the same certified cardiovascular surgeons and anesthetists who were well trained to manage patients undergoing cardiovascular surgery, and the surgical outcomes were stable during the present study period. Although the study period extended for 5 years, the changes in surgical and anesthetic management during the period were very minor and are unlikely to impact the present results. In addition, the small differences in rSO_2_ values between patients with and without CAS are not likely to be of clinical importance even if the sample size were large enough to possibly detect statistically significance. In the present study, neurological complication was defined as focal neurologic deficit persisting ≥ 24 h and confirmed by brain CT or MRI. However, it is difficult to evaluate neurological outcomes of patients with stroke and/or TIA in anamnesis. Thus, the present study should have had to enroll only patients without neurological issues. Furthermore, since cardiac index was estimated by thermodilution technique with a pulmonary artery catheter in the present study, cardiac index in the presence of tricuspid regurgitation, such as during grafting the LCX or RCA, might be inaccurate. However, transesophageal echocardiography is not useful in such situations since the contact between the esophagus and pericardium is interrupted by air. Although SvO_2_, which is known to be independent of tricuspid regurgitation, is a useful hemodynamic parameter for estimating cardiac output, missing SvO_2_ values were too much to confirm our results. Thus, the presence of tricuspid regurgitation was not assessed in the present study. However, consistent with a previously reported study [27], decreases in cardiac index was identified to be associated with decreases in rSO_2_ during off-pump CABG in adult patients. Further prospective studies including postoperative cognitive dysfunction assessment are warranted to confirm our findings.

## Conclusions

In the present retrospective study, cerebral oxygenation significantly decreased during anastomosis in both patients with and without CAS, but CAS was not associated with an increased risk of a decrease in rSO_2_ value ≥ 10 % from preoperative value during anastomosis. In addition, a decrease in cardiac index, but not a decrease in MAP and an increase in CVP, was associated with a decrease in rSO_2_ value ≥ 10 % from preoperative value during anastomosis in both patients with and without CAS. In patients with CAS as well as those without CAS, maintaining cardiac output, as compared to increasing MAP, appears to be a better strategy for preventing a decrease in cerebral oxygenation during off-pump CABG.

### Consent

The Institutional Ethics Committee waived the requirement for written informed consent due to the retrospective design of the study.

## Abbreviations

CABG: Coronary artery bypass grafting
CPB: cardiopulmonary bypass (CPB)
LVEF: Left ventricular ejection fraction
CAS: Carotid artery stenosis
NIRS: Near-infrared spectroscopy
rSO_2_: Regional cerebral tissue oxygen saturation
CT: Computed tomography
ETCO_2_: End-tidal carbon dioxide
BIS: Bispectral index
CVP: Central venous pressure
SvO_2_: mixed venous blood oxygen saturation
MAP: Mean arterial pressure
LAD: left anterior descending coronary artery
LCX: left circumflex coronary artery
RCA: right coronary artery
MRI: magnetic resonance imaging
ANOVA: Analysis of variance
OR: Odds ratio
CI: Confidence interval
TIA: Transient ischemic attack
SPECT: Single photon emission computed tomography
CMRO_2_: Cerebral metabolic rate of oxygen
CBF: Cerebral blood flow
